# Spatiotemporally controlled generation of NTPs for single-molecule studies

**DOI:** 10.1038/s41589-022-01100-9

**Published:** 2022-09-21

**Authors:** Anton Sabantsev, Guanzhong Mao, Javier Aguirre Rivera, Mikhail Panfilov, Anatolii Arseniev, Oanh Ho, Mikhail Khodorkovskiy, Sebastian Deindl

**Affiliations:** 1 Department of Cell and Molecular Biology, Science for Life Laboratory, Uppsala University, Uppsala, Sweden; 2 Peter the Great St. Petersburg Polytechnic University, Saint Petersburg, Russia; 3 Center for Precision Genome Editing and Genetic Technologies for Biomedicine, Institute of Gene Biology, Russian Academy of Sciences, Moscow, Russia

**Keywords:** Single-molecule biophysics, Biophysical chemistry

## Abstract

Many essential processes in the cell depend on proteins that use nucleoside triphosphates (NTPs). Methods that directly monitor the often-complex dynamics of these proteins at the single-molecule level have helped to uncover their mechanisms of action. However, the measurement throughput is typically limited for NTP-utilizing reactions, and the quantitative dissection of complex dynamics over multiple sequential turnovers remains challenging. Here we present a method for controlling NTP-driven reactions in single-molecule experiments via the local generation of NTPs (LAGOON) that markedly increases the measurement throughput and enables single-turnover observations. We demonstrate the effectiveness of LAGOON in single-molecule fluorescence and force spectroscopy assays by monitoring DNA unwinding, nucleosome sliding and RNA polymerase elongation. LAGOON can be readily integrated with many single-molecule techniques, and we anticipate that it will facilitate studies of a wide range of crucial NTP-driven processes.

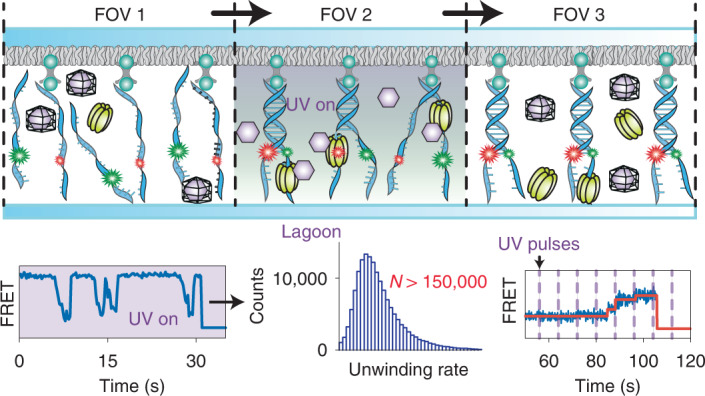

## Abstract (web summary)

A new method for controlling NTP-driven reactions in single-molecule experiments via the local generation of NTPs (LAGOON) markedly increases the measurement throughput and enables single-turnover observations.

## Main

Life is an inherently non-equilibrium process, and the study of critical cellular functions requires observations of irreversible processes. Many of these processes, including genome transactions as well as cellular signaling and transport, involve NTP-utilizing enzymes. These enzymes encompass polymerases, molecular motors, transport proteins and signaling switches, and they often exhibit complex dynamic behaviors. The study of such intricate dynamics is greatly facilitated by single-molecule methods, because they avoid ensemble averaging, which often obscures transient kinetic intermediates and alternative kinetic pathways^1–14^. However, virtually all NTP-hydrolyzing enzymes irreversibly alter the biological macromolecules that they act upon. Because optical trapping, magnetic tweezers or fluorescence measurements are often conducted on surface-immobilized substrates, this substantially limits the measurement throughput. Such experiments are typically initiated by perfusing the flow cell with enzymes and/or NTP cofactors. Per experiment, only one molecule or a small area of the flow cell surface corresponding to a field of view (FOV) can, thus, be imaged, because, after data acquisition, the reaction has already taken place everywhere else on the surface of the flow cell. Consequently, the reaction can be observed for a small fraction of the substrates only. In a typical single-molecule fluorescence resonance energy transfer (smFRET) experiment, fewer than 2,000 molecules can be observed in a single FOV, a number that can be increased to around 15,000 by using individual sCMOS cameras for each channel^15^. In force spectroscopy experiments, a throughput of hundreds can be achieved with magnetic tweezers^16^ and acoustic force spectroscopy (AFS)^17^, which can be further extended to around 10,000 at the cost of substantially lower resolution using flow^18^. Furthermore, many NTP-driven processes involve complex intermediates that are formed over multiple sequential turnovers. Dissecting the properties of such intermediates without the precise temporal control of NTP availability remains challenging. Here we present a new method that enables the observation of irreversible NTP-driven processes in many FOVs of the same experiment, thereby completely removing a fundamental limitation in the throughput of single-molecule measurements. At the same time, the precise spatiotemporal control over NTP availability offered by our method greatly facilitates the dissection of reaction intermediates and enables single-turnover observations at the single-molecule level.

## Results

### Controlling helicase unwinding in a smFRET assay with LAGOON

**Fig. 1:**
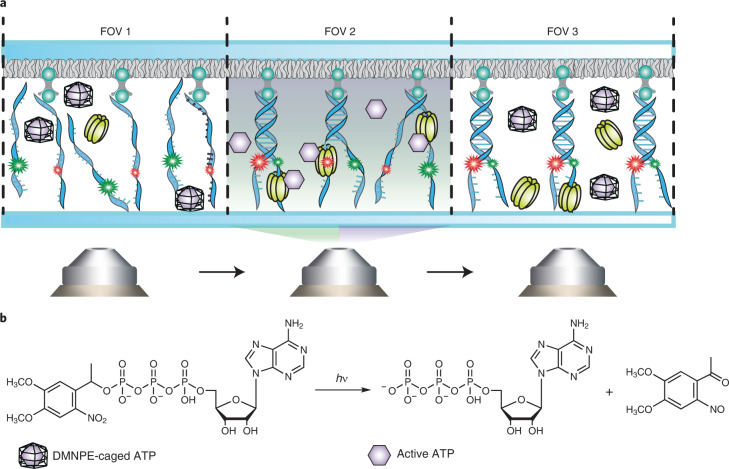
The concept of spatiotemporal control over NTP-driven reactions via LAGOON. **a**, Schematic of a LAGOON experiment monitoring the helicase-catalyzed unwinding of DNA as an example, depicting FOVs after (FOV 1), during (FOV 2) and before (FOV 3) ATP uncaging. Y-shaped DNA constructs with Cy3 (green) and Cy5 (red) fluorophores attached at the beginning of the duplex region are immobilized on the surface via streptavidin–biotin interactions with biotinylated PEG. Unwinding of the DNA duplex by the T4 helicase results in the decrease of the initially high FRET. **b**, Structure and uncaging of DMNPE-caged ATP.

**Fig. 2:**
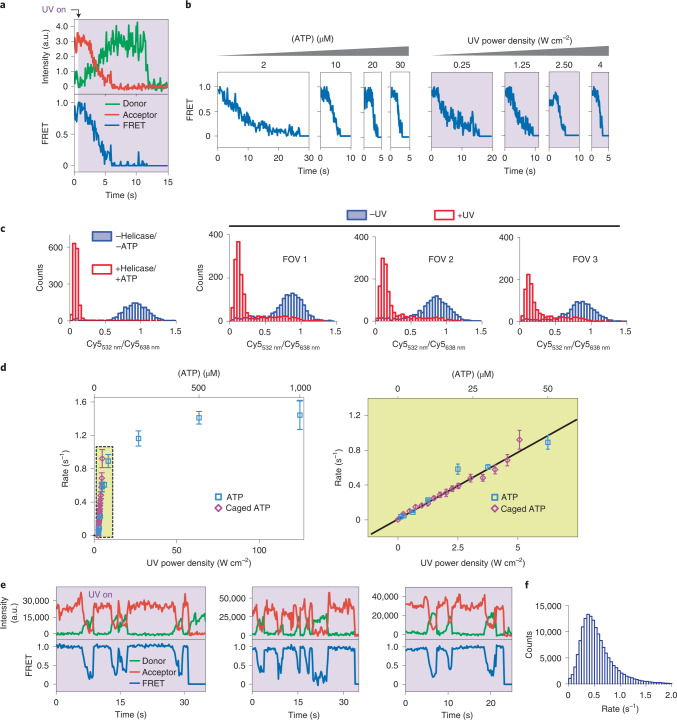
smFRET observations of DNA unwinding by T4 helicase using LAGOON. **a**, Representative donor (green), acceptor (red) and FRET time traces (out of *n* = 117 traces) showing the unwinding of a single DNA molecule upon continuous ATP uncaging with 4 W cm^−^^2^ UV laser power density (purple). **b**, Representative FRET time traces showing the unwinding of individual DNA molecules at different ATP concentrations in a standard experiment (left, *n* = 775, 1,388, 145 and 441) or at different uncaging laser intensities in a LAGOON experiment with continuous uncaging (right, *n* = 85, 178, 153 and 303). **c**, Histograms of the ratio between Cy5 fluorescence intensities upon 532-nm and 638-nm excitation before and after unwinding in a standard experiment (left, *n* = 1,513) or before and after continuous uncaging for 1 minute with 0.75 W cm^−^^2^ UV laser power density in three FOVs on the same slide in a LAGOON experiment with 10 mM DMNPE-ATP (right, *n* = 1,454, 1,327 and 1,106). The Cy5_532nm_/Cy5_638nm_ ratio was chosen instead of FRET because the donor-labeled DNA strand dissociated upon unwinding, leading to the absence of any detectable FRET signal. **d**, DNA unwinding rates (mean ± s.e.m.) as a function of ATP concentration (*n* = 637, 775, 1,800, 1,388, 145, 441, 182, 222, 209 and 131) or uncaging laser power density in the presence of 2 mM DMNPE-ATP (*n* = 85, 141, 134, 169, 178, 252, 104, 94, 186, 153, 210, 305, 303, 229 and 85) upon continuous uncaging. The plot on the right represents an enlarged version of the region marked in yellow in the plot on the left, scaled to overlay the linear sections of two titration curves. **e**,**f**, High-throughput smFRET observations of DNA unwinding using LAGOON on a motorized objective-type TIRF setup. **e**, Representative FRET time traces showing the unwinding of individual DNA molecules during continuous uncaging with approximately 2.5 W cm^−^^2^ 405-nm laser in the presence of 200 nM T4 helicase, 10 mM DMNPE-ATP and 10 U ml^−1^ of hexokinase (out of *n* = 64,243 traces from 112 FOVs). **f**, Histogram of DNA unwinding rates (*n* = 158,523 unwinding events). The experiments (**e**,**f**) were carried out on a Nikon Eclipse Ti2-E motorized microscope equipped with a CFI Apochromat TIRF 60XC objective. a.u., arbitrary unit.

**Extended Data Fig. 1:**
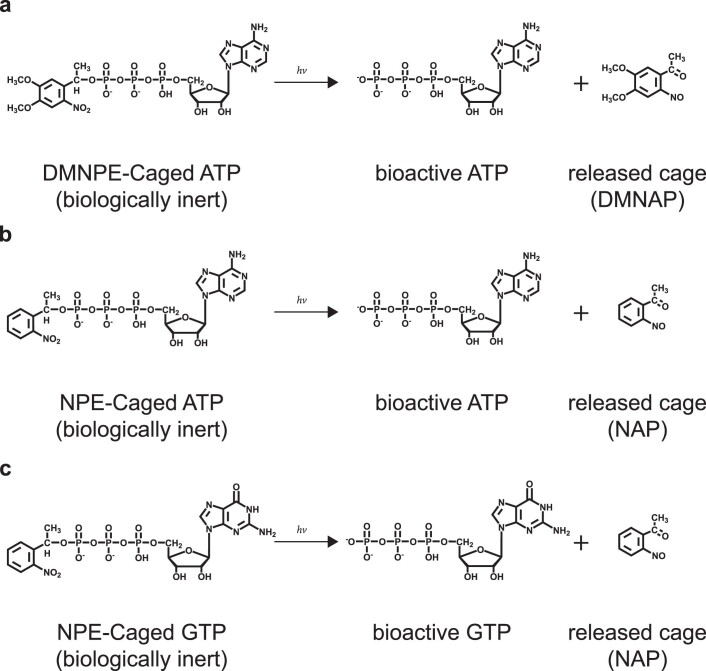
Caged NTP compounds used in the experiments. Chemical structures and uncaging of DMNPE-ATP (**a**), NPE-ATP (**b**), and NPE-GTP (**c**). DMNPE-ATP and NPE-GTP were used in smFRET LAGOON experiments. NPE-ATP was used in AFS LAGOON experiments.

**Extended Data Fig. 2:**
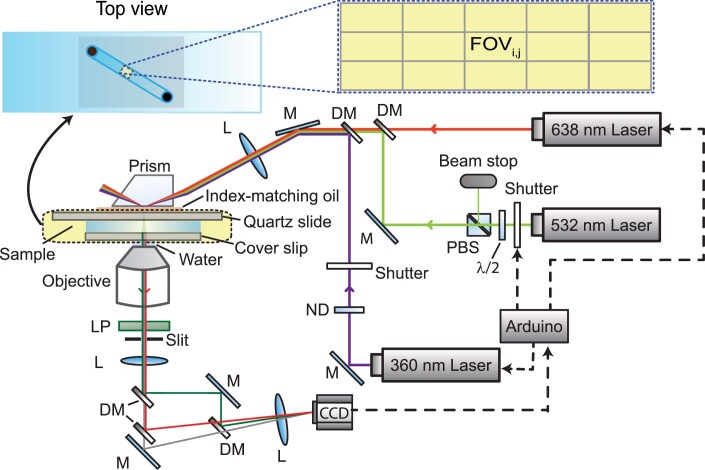
Schematic of the smFRET optical setup adapted for LAGOON experiments. DM = dichroic mirror, PBS = polarization beam splitter, λ/2 = half-wave plate, M = mirror, L = lens, LP = long-pass filter, ND = neutral density filter. See Methods for a detailed description of the setup.

We reasoned that both the measurement throughput as well as the analysis of transient kinetic intermediates could be greatly enhanced if NTPs could be made available on demand. To this end, we substituted the NTP substrate for its caged analog^19^ (Fig. 1 and Extended Data Fig. 1). In this analog, the terminal phosphate is esterified with a blocking group, which renders the molecule biologically inactive. The free nucleotide is released only upon photolysis of the blocking group. Such compounds have previously enabled ensemble experiments with myosin^20^ and RNA polymerase (RNAP)^21^, among others^22–27^. To demonstrate the utility of caged NTPs for high-throughput measurements of irreversible NTP-driven transactions at the single-molecule level, we incorporated a 360-nm uncaging laser into a total internal reflection fluorescence microscope and turned to a well-established smFRET helicase assay^28–30^ (Fig. 1a and Extended Data Fig. 2). This assay involves a Y-shaped DNA construct, where donor and acceptor fluorophores are attached to opposing strands at the beginning of the duplex region. Consequently, duplex unwinding causes a decrease in FRET and, eventually, the complete dissociation of the donor-labeled strand. The reaction is initiated by the addition of helicase and ATP, which allows for data acquisition within one FOV only, because, thereafter, the reaction has already taken place everywhere on the surface of the flow cell. We reasoned that it should be possible to trigger the reaction on demand within a confined area of the slide by using a UV laser to release free ATP only within the chosen FOV (Fig. 1a). For our method, the local generation of NTPs (LAGOON), to work as intended, free ATP must not be present before activation, and the diffusion of free ATP upon activation must be limited. We, therefore, supplemented the flow cell with the ATP-consuming enzyme hexokinase at a concentration sufficiently low to permit unwinding in the observed FOV yet sufficiently high to suppress the diffusion of uncaged ATP into neighboring FOVs. Indeed, a reaction containing T4 helicase, caged ATP and hexokinase allowed unwinding only when and where the activating laser was on (Fig. 2a,c and Supplementary Fig. 1). Thus, dozens and even hundreds of FOVs can be imaged on the same slide, thereby markedly increasing the measurement throughput. Moreover, because the concentration of available ATP is proportional to the intensity of the uncaging laser, LAGOON provides a highly convenient means to control the reaction speed. We note that the photodamage and fluorophore photobleaching induced by the uncaging laser is negligible for moderate intensities. At a power density of ~10 W cm^−^^2^ (twice the maximum power density used for the helicase experiments in this work), the photobleaching half-times are approximately 90 seconds for Cy5 and 180 seconds for Cy3 (Supplementary Fig. 2). In terms of photodamage, ~63% of DNA molecules were successfully unwound in the presence of 20 nM T4 helicase and 1 mM ATP even after a 1-minute exposure to the 360-nm light at ~10 W cm^−^^2^ in the presence of 1 mM DMNPE-ATP. Furthermore, to assess the reproducibility of LAGOON measurements, we conducted helicase experiments on three FOVs in three different flow cells with ~1 W cm^−^^2^ 360-nm laser power density and quantified the rates of FRET change during unwinding. The relative standard deviation for individual FOVs within the flow cell was 6% and 13% between individual flow cells (*n* = 821, 553, 545, 545, 450, 249, 461, 455 and 528 unwinding events in each FOV, respectively). The variation of the unwinding extent across the FOV was <12% (Supplementary Fig. 3). LAGOON, therefore, enables, for example, the near-effortless acquisition of data for a detailed ATP titration curve from many FOVs of a single microscopy slide (Fig. 2b,d). The effective concentration of free ATP at a given uncaging laser intensity can be readily obtained from a comparison of the ATP titration curves with regular and caged ATP (Fig. 2d). Under these conditions, an effective ATP concentration of up to 40 µM could be achieved. By increasing the concentration of caged ATP and decreasing that of hexokinase, the effective ATP concentration can be increased approximately six-fold (Supplementary Fig. 4). Thus, an ATP concentration of at least 250 µM can be obtained at the maximum 360-nm uncaging power density used in this study. Although readily achievable in the prism-type total internal reflection fluorescence (TIRF) configuration, commercially available TIRF setups rely on objective-type TIRF microscopy instead of the prism-based TIRF imaging that we used to establish LAGOON. Because most TIRF objectives have very low transmittance at 360 nm, we also tested the ability to perform LAGOON smFRET experiments with 405-nm uncaging on an objective-type TIRF setup. We were able to observe DNA unwinding by the T4 helicase in an smFRET assay on a commercially available objective-type TIRF microscope (Fig. 2). At an uncaging power density of 2 W cm^−^^2^ with 10 mM DNMPE-ATP and 1 U ml^−1^ of hexokinase, the unwinding rate was 0.26 ± 0.02 s^−1^ (*n* = 126 unwinding events), which corresponds to an effective ATP concentration of ~12.5 µM. The maximum uncaging power density that we found to be compatible with efficient smFRET imaging was 10 W cm^−^^2^. We, therefore, estimate that effective ATP concentrations over 50 µM can be readily achieved in LAGOON smFRET experiments with 405-nm uncaging. We additionally characterized the diffusion of uncaged ATP into neighboring regions. The extent of unwinding decreased by two-fold at a distance of ~7 µm from the edge of the area illuminated with the 405-nm uncaging laser (Supplementary Fig. 1), indicating that ATP can diffuse to, at most, a distance of several tens of microns into neighboring areas. ATP diffusion, therefore, does not pose a substantial limitation on the throughput of LAGOON experiments, because several square centimeters of flow cell area are typically available for imaging. Moreover, this motorized microscope enabled us to perform automated LAGOON data acquisition on >100 FOVs, resulting in >60,000 DNA unwinding traces capturing >150,000 unwinding events (Fig. 2f). LAGOON, thus, markedly enhances the throughput for observations of irreversible NTP-driven processes at the single-molecule level.

### Single-turnover observations of nucleosome sliding

**Fig. 3:**
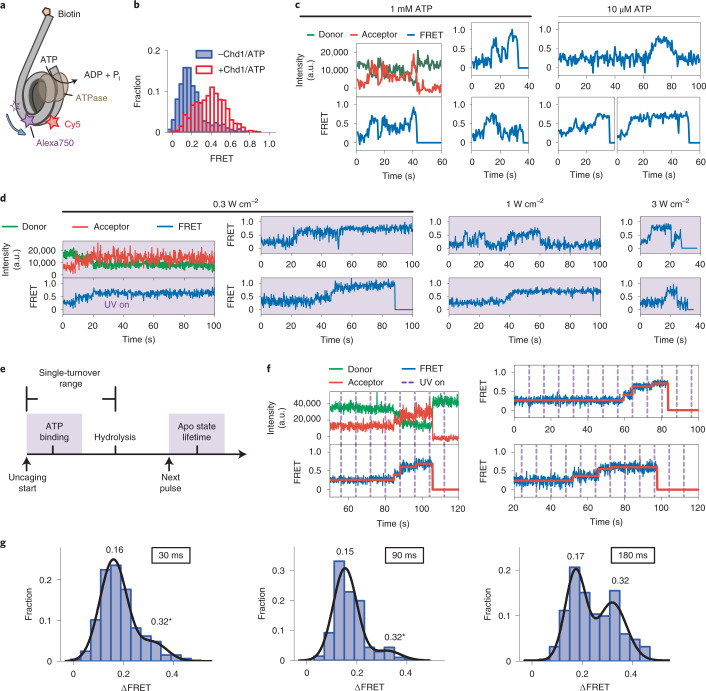
Single-turnover observations of remodeling with LAGOON. **a**, Representation of the labeling scheme for observing the DNA movement at the entry side during nucleosome sliding. The Cy5 fluorophore (red) attached to the exit-side H2B-120C is excited with the 638-nm laser and acts as a FRET donor, whereas the Alexa750 fluorophore (purple) attached to the entry-side DNA acts as a FRET acceptor. **b**, FRET histograms before (*n* = 855) and after (*n* = 726) the addition of Chd1 and 1 mM ATP. **c**, Representative donor (green), acceptor (red) and FRET (blue) time traces showing the remodeling of individual nucleosomes by Chd1 in the presence of 1 mM (left, *n* = 145) or 10 µM (right, *n* = 89) ATP. **d**, Representative donor (green), acceptor (red) and FRET (blue) time traces showing the remodeling of individual nucleosomes by Chd1 in a LAGOON experiment in the presence of 10 mM DMNPE-ATP with 0.30 W cm^−^^2^ (left, *n* = 93), 1 W cm^−^^2^ (middle, *n* = 91) or 3 W cm^−^^2^ (right, *n* = 72) continuous uncaging. **e**, A simplified schematic of the single-turnover experiment. Average times are marked for stochastic processes. **f**, Representative donor (green), acceptor (red) and FRET (blue) time traces showing entry-side DNA movement during nucleosome sliding in the single-turnover regime in the presence of 10 mM DMNPE-ATP (*n* = 209). The purple dashed lines mark uncaging laser pulses (90-ms duration every 8 seconds, 100 W cm^−^^2^ uncaging power density). Red lines in the FRET time traces represent the steps identified in the data using the AutoStepFinder method^48^. **g**, Histograms of FRET change in the first observed step upon pulsed ATP uncaging with a pulse duration of 30 ms (top, *n* = 453), 90 ms (middle, *n* = 206) or 180 ms (bottom, *n* = 254). Black lines represent fits of the histograms with two Gaussian peaks. Pulse period, 8 seconds. The asterisk indicates that, for the second peak, its center and width were fixed for the 30-ms and 90-ms histograms to match the parameters derived from the second peak of the 180-ms histogram. a.u., arbitrary unit.

Apart from the spatial control over the reaction onset, LAGOON offers the precise temporal control over NTP availability. By providing NTP in pulses that are shorter than the average duration of hydrolysis, it should be possible to achieve conditions where, at most, a single NTP molecule can be hydrolyzed per pulse (Fig. 3e). Achieving such control over individual NTP turnover events would represent a powerful tool for dissecting particularly complex molecular mechanisms. A good example is nucleosome sliding catalyzed by ATP-dependent chromatin remodeling enzymes (remodelers)^31–37^, where several unstable nucleosome intermediates are formed during multiple consecutive ATP hydrolysis cycles before a nucleosome is stably repositioned^38,39^. For example, the Chd1 (chromodomain helicase DNA-binding protein 1) remodeler ATPase pulls up to four DNA base pairs into the entry side of the nucleosome, forming transient nucleosome structures that buffer the additional DNA, before it is released at the opposite (exit) side^38^. We, therefore, used an smFRET assay that monitors the movement of the entry-side nucleosomal DNA during nucleosome sliding by Chd1 (refs. ^38,40^) (Fig. 3a). In this assay, the donor is attached to histone H2B and the acceptor dye to the DNA at the entry side of the nucleosome, such that, upon remodeling, the initially low FRET increases as the entry-side DNA is moved into the nucleosome (Fig. 3a–c). During continuous remodeling, the acceptor eventually passes the donor, and FRET decreases again, which cannot be unambiguously distinguished from non-processive remodeling or a reversal in remodeling direction^38^. We, therefore, limited our analyses to the initial phase of monotonous FRET increase.

**Extended Data Fig. 3:**
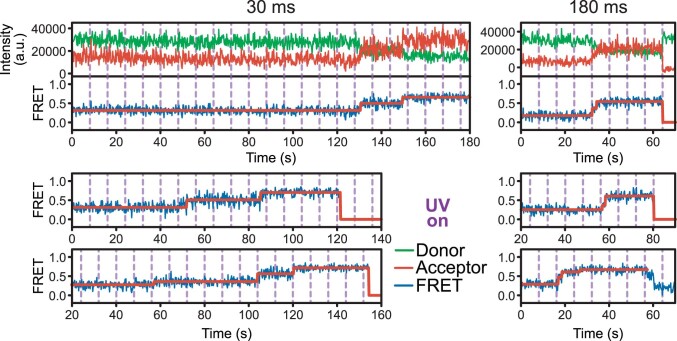
Nucleosome sliding by Chd1 observed using LAGOON with pulsed uncaging. Representative donor (green), acceptor (red), and FRET (blue) time traces showing the remodeling of individual nucleosomes by Chd1 in a LAGOON experiment with pulsed uncaging in the presence of 10 mM DMNPE-ATP. Pulse duration 30 ms (left, *N* = 454) or 180 ms (right, *N* = 333), pulse period 8 s, uncaging power density 100 W/cm^2^. Red lines in the FRET time traces represent the steps identified using the AutoStepFinder method^60^.

**Extended Data Fig. 4:**
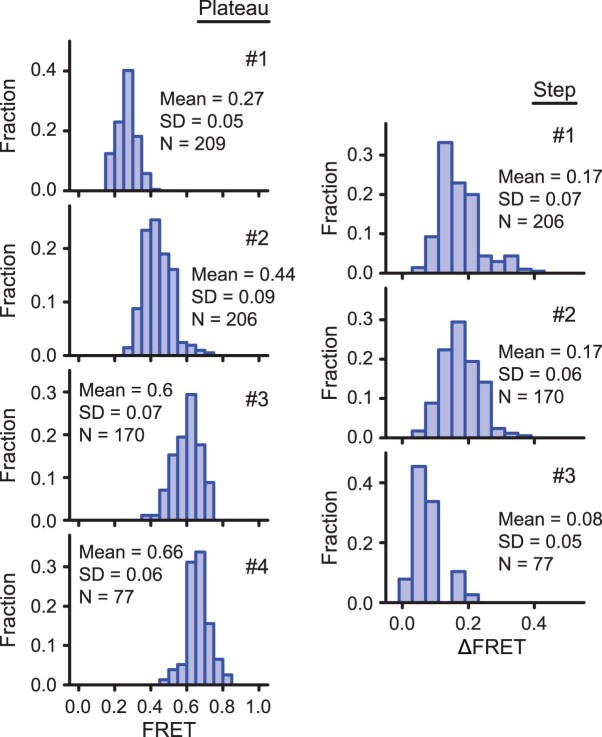
Characterization of remodeling intermediates observed with LAGOON in the single-turnover regime. Histograms of four consecutive FRET plateaus (left) and corresponding FRET changes (right) in time traces (*N* = 209) showing the remodeling of individual nucleosomes by Chd1 in a LAGOON experiment with pulsed uncaging in the presence of 10 mM DMNPE-ATP. Pulse duration 90 ms, pulse period 8 s, uncaging power density 100 W/cm^2^.

Analogous to the helicase reaction, the onset and speed of remodeling could be controlled using LAGOON with continuous uncaging (Fig. 3d), allowing for high-throughput observations. To implement single-turnover conditions, the uncaging pulse must be sufficiently long to permit ATP binding yet sufficiently short to minimize the probability of multiple ATP turnovers per pulse. Moreover, the pulse frequency must be sufficiently high to ensure the processive build-up of unstable intermediates yet sufficiently low to enable the completion of ATP turnover and the observation of intermediates. Because the average ATP hydrolysis time for Chd1 is around 200–600 ms^41,42^, we tested pulse durations of 30–180 ms (see also Supplementary Note 1 and Supplementary Fig. 5 for numerical simulations of uncaging). A pulse duration of 30 ms resulted in many non-processive traces, whereas a pulse duration of 180 ms often led to compound steps, which is apparent in the distribution of FRET changes observed for the first step (Fig. 3g and Extended Data Fig. 3). The distribution is bimodal in the case of 180-ms pulses, with over 45% of traces exhibiting a larger, compound step size, whereas, for both 90-ms and 30-ms pulses, this fraction is below 16%. Notably, despite a marked decrease in processivity, the size of the predominant FRET change for a pulse duration of 30 ms is the same as that observed for 90 ms, confirming that this FRET change corresponds to the elementary step of the reaction. For this reason, we chose a pulse duration of 90 ms, providing an optimal balance between processivity and the strictness of the single-turnover regime (Fig. 3f,g). Strikingly, our pulsed LAGOON experiments enabled single-turnover observations at the single-molecule level and allowed us to resolve at least three consecutive ATP hydrolysis steps and their corresponding, as-of-yet uncharacterized remodeling intermediates (Fig. 3f and Extended Data Fig. 4). Despite the fact that continuous uncaging experiments could also capture such intermediates (Fig. 3d), their observation in the single-turnover regime allowed resolving them in much greater detail and substantially facilitated data interpretation (for example, identification of the elementary steps of the reaction). LAGOON, therefore, provides unique capabilities to probe and resolve reaction intermediates in NTP-driven processes.

### Controlling an RNAP with LAGOON

**Fig. 4:**
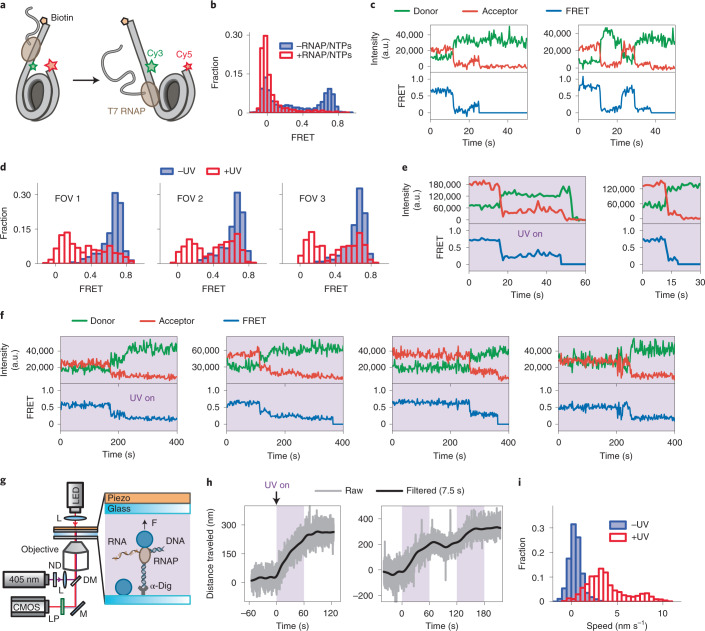
Observations of RNAP activity with LAGOON. **a**, Representation of the labeling scheme for observing nucleosome unwrapping upon its invasion by the T7 RNAP. **b**, FRET histograms before (*n* = 1,667) and after (*n* = 1,759) the addition of the T7 RNAP and NTPs. **c**, Representative donor (green), acceptor (red) and FRET (blue) time traces (*n* = 294) showing the unwrapping of individual nucleosomes by the T7 RNAP in the presence of NTPs (0.5 mM each). **d**, FRET histograms from three FOVs on the same slide (*n* = 349, 256 and 233, respectively) before and after uncaging for 2 minutes with 10 W cm^−^^2^ UV laser power in the presence of 2 mM DMNPE-ATP and CTP, GTP and UTP (0.5 mM each). The presence of the acceptor fluorophore (Cy5) was, in this case, probed by direct excitation with the 638-nm laser, which allowed the constructs lacking the acceptor, forming a low-FRET population in the absence of RNAP activity in **b**, to be excluded from the analysis. **e**, Representative donor (green), acceptor (red) and FRET (blue) time traces (*n* = 156) showing the unwrapping of individual nucleosomes by the T7 RNAP in a LAGOON experiment upon continuous uncaging with 10 W cm^−^^2^ UV laser power in the presence of 2 mM DMNPE-ATP and CTP, GTP and UTP (0.5 mM each). **f**, Representative donor (green), acceptor (red) and FRET (blue) time traces showing the unwrapping of individual nucleosomes by the T7 RNAP in a LAGOON experiment upon continuous uncaging with 3 W cm^−^^2^ UV laser power in the presence of 1 mM NPE-GTP, CTP, ATP and UTP each (*n* = 212). **g**, Schematic of the AFS LAGOON experiment monitoring *E. coli* RNAP elongation. DM, dichroic mirror; L, lens; LP, long-pass filter. See Methods for a detailed description of the setup. **h**, Representative raw (gray) and smoothed (with a fast Fourier transform filter, black) RNAP elongation time traces upon intermittent uncaging (*n* = 11 trajectories). The purple background represents ATP uncaging with a UV laser (~25 mW cm^−^^2^). **i**, Histograms of RNAP elongation rates with or without uncaging. The reaction buffer was supplemented with 1 mM NPE-ATP, CTP, GTP and UTP each. a.u., arbitrary unit.

To illustrate the broad utility of our method, we used LAGOON to examine the complex NTP-driven process of transcription, where an RNAP uses the NTPs as building blocks by transferring their nucleotidyl moiety to the hydroxyl at the 3′ terminus of the nascent transcript. Many single-molecule techniques have been applied with great success to the study of transcription^7–11^. To explore whether LAGOON could facilitate the study of this key cellular process, we designed an smFRET assay that monitors the invasion of a nucleosome by the transcribing RNAP^43–45^. As a proof of concept, we used a commercially available T7 RNAP. We reconstituted nucleosomes where both the FRET donor (Cy3) and acceptor (Cy5) fluorophores are attached to the nucleosomal DNA, initially close to each other. The long DNA linker on one side of the nucleosome contains the T7 promoter sequence. During transcription, the RNAP induces the unwrapping of DNA from the nucleosome. As a consequence, donor and acceptor dyes become separated, leading to a FRET decrease (Fig. 4a–c). We reasoned that, for a transcription reaction containing T7 RNAP, hexokinase and caged ATP, as well as free UTP, CTP and GTP, LAGOON should enable spatiotemporal control via the availability of free ATP. Indeed, the LAGOON approach allowed for the transcription reaction to be initiated at will in a confined area of the slide (Fig. 4d,e). Replacing GTP rather than ATP with its caged analog enabled similar control over the transcription reaction (Fig. 4f). The LAGOON approach can, thus, be applied to single-molecule investigations of transcription.

### Combining LAGOON with force spectroscopy

**Extended Data Fig. 5:**
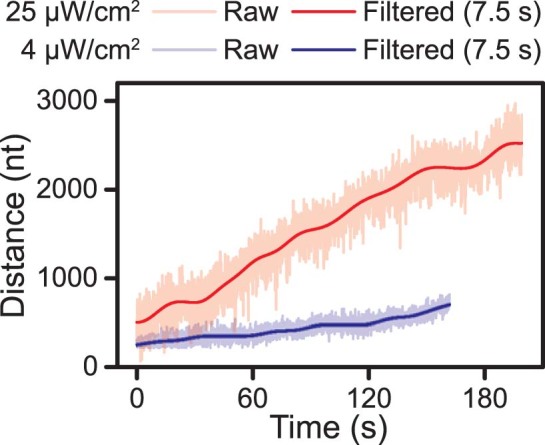
E. coli RNAP elongation as a function of unaging power density in an AFS LAGOON experiment. Representative raw (transparent) and smoothed (with a fast Fourier transform filter, solid) RNAP elongation time traces upon uncaging with ~25 µW/cm^2^ UV laser (red, *N* = 11) or ~4 µW/cm^2^ (blue, *N* = 10).

To further demonstrate the general applicability of our method, we sought to integrate LAGOON with force spectroscopy, another powerful family of single-molecule methods. As an example, we tested LAGOON in combination with AFS to monitor transcription elongation^17,46^ (Fig. 4g–i and Extended Data Fig. 5). In this assay, one end of the DNA substrate is attached to the surface of the AFS chip. The biotinylated *Escherichia coli* RNAP is linked to a streptavidin-coated microsphere held under a constant opposing force. Transcription results in the movement of the microsphere toward the surface of the chip, which is monitored by light microscopy. To implement LAGOON, we modified the AFS setup to incorporate an uncaging UV laser (Fig. 4g) and replaced ATP in the nucleotide mixture with its caged analog. Under these conditions, we observed elongation only when the uncaging laser was turned on (Fig. 4h,i), with higher uncaging intensities producing increased elongation rates (Extended Data Fig. 5). At the higher uncaging intensity, tether half-life was around 9 minutes, indicating that photodamage caused by UV illumination was not significantly limiting. LAGOON can, therefore, be used to control complex NTP-dependent reactions in force spectroscopy experiments.

## Discussion

Despite great success in the study of NTP-driven processes by single-molecule methods, such experiments are substantially constrained by the lack of precise control over NTP availability in space and time. By employing caged NTP analogs, we developed a new technique (LAGOON) that overcomes these limitations and substantially expands the possibilities of single-molecule analysis as applied to this important class of irreversible processes. As an example, we demonstrated the ability to achieve high-throughput observations using DNA unwinding by a hexameric helicase. Next, we applied LAGOON to the intricate and as-of-yet incompletely understood process of remodeler-catalyzed nucleosome sliding. By providing ATP in short pulses, we were able to achieve single-turnover observations spanning multiple consecutive translocation steps. This development allowed us to detect previously uncharacterized kinetic intermediates during nucleosome remodeling, highlighting the power of temporal control over NTP-driven reactions provided by LAGOON. We further demonstrated the applicability of LAGOON to the study of the complex process of transcription using both smFRET and force spectroscopy, where we achieved spatiotemporal control over the reaction progress. We anticipate that this approach will greatly facilitate future studies of transcription at the single-molecule level. Other photocaged ligands could prove useful for LAGOON-type experiments, including caged cAMP, NAD, NADP, photolabile divalent ion chelators (for example, ‘caged Ca’) and many others. Indeed, our preliminary data indicate that DMNP-EDTA (‘caged Ca’) can be used to control the DNA helicase reaction in an smFRET experiment (Supplementary Fig. 6), offering a more general approach for controlling reactions that require divalent ions.

We note that, for both smFRET and AFS, the modifications required to implement LAGOON were minimal. In combination with the commercial availability of various different caged NTPs, this implies that LAGOON can be readily and cost-efficiently integrated with most existing single-molecule instruments. In a typical smFRET experiment, LAGOON enables data acquisition for >100,000 molecules, in >100 FOVs of a single flow cell with >1,000 molecules per FOV. By extending the total imaging time, >1,000,000 observations can be achieved (for example, 1,000 FOVs can be observed for 1 minute each in under 17 hours). Combining LAGOON with methods that increase the number of molecules observed in each FOV would additionally speed up the acquisition of very large datasets^15,18^. In principle, LAGOON could make it possible to image most of the >50 million molecules in a typical smFRET flow cell. At the other end of the spectrum, extremely high temporal resolution can be achieved when monitoring a single molecule only in an smFRET or force spectroscopy experiment. However, the inherently low throughput of such experiments imposes challenging limitations for the study of non-equilibrium processes. By overcoming these limitations, LAGOON renders such high-resolution observations viable and opens exciting new opportunities for the study of NTP-driven processes with high temporal and spatial resolution. Optical trapping experiments in a dumbbell configuration offer control over buffer composition by moving the substrate between different channels in a laminar flow cell, which can be used to control NTP availability. However, moving between different channels takes substantially longer when compared to LAGOON, and the throughput of such experiments is limited due to the time it takes to assemble the dumbbell. We envision LAGOON achieving precise control over NTP availability in dumbbell optical trapping experiments (for example, to achieve a single-turnover regime) and to markedly increase the throughput of optical trapping experiments with surface-tethered substrates. Moreover, coumarin-based^21^ and new thiocoumarin-based^47^ caged compounds with nanosecond-scale uncaging times and longer uncaging wavelengths that cause less photodamage will likely be useful for force spectroscopy–based LAGOON experiments.

To summarize, we developed a broadly applicable technique, LAGOON, for achieving spatiotemporal control over NTP-dependent processes in single-molecule experiments. By allowing sequential observations of irreversible transactions in hundreds of FOVs on a single microscopy slide, LAGOON enables high-throughput single-molecule observations of NTP-driven processes. We anticipate that LAGOON will enable the observation of rare events and greatly increase the spatial and temporal resolution of single-molecule fluorescence experiments, where resolution is determined by a balance between photobleaching and the number of observations. By offering precise temporal control over NTP availability, LAGOON greatly facilitates the interrogation of intermediates formed over the course of multiple sequential turnovers in complex NTP-driven processes. Given that our method requires only the straightforward addition of an uncaging UV laser, we anticipate that LAGOON will be applied to most single-molecule techniques and facilitate mechanistic studies of a wide range of critical NTP-utilizing enzymes in transcription, translation, replication, DNA repair, cellular signaling and transport processes.

## Methods

### Enzymes

T4 helicase, T7 RNAP and *Saccharomyces cerevisiae* hexokinase were purchased from MCLab (T4DH), New England Biolabs (NEB, M0251) and Sigma-Aldrich (H4502), respectively. Hexokinase was dissolved in 10 mM Tris-HCl pH 8.0 buffer with 50 mM NaCl at a concentration of 1,000 U ml^−1^ (based on the information provided by the manufacturer), aliquoted and stored at −20 °C. A new aliquot of hexokinase was used for each experimental day and kept on ice after thawing. The pIA497 co-overexpression plasmid (gift from I. Artsimovitch) encoding the biotin carboxyl carrier protein at the end of the β′ *E. coli* RNAP subunit was used to obtain the biotinylated *E. coli* RNAP core enzyme as described previously^46^. The plasmid was transformed into *E. coli* BL21 cells, which were grown to OD 0.6 and then induced with 1 mM imidazole for 6 hours at 37 °C. After centrifugation, pellets were resuspended in lysis buffer A (40 mM Tris-HCl pH 8, 500 mM NaCl, 1 mM DTT, 0.5 mM EDTA, 10% glycerol) supplemented with 5 mM imidazole and 1 µg ml^−1^ of lysozyme and sonicated. Next, the lysate was applied to a 1-ml HisTrap HP column (GE Healthcare) and, after several washes, eluted with buffer A supplemented with 300 mM imidazole. The resulting fractions were concentrated and applied to a Superose 6 (GE Healthcare) column. Peak fractions were purified by anion-exchange chromatography using a 1-ml monoQ anion-exchange column (GE Healthcare). Fractions corresponding to the core enzyme were concentrated and stored frozen at −80. The *E. coli* RNAP σ70 subunit was purified from overexpressing cells by affinity (Ni-NTA) and size-exclusion chromatography as described previously^49^. The RNAP holoenzyme was obtained by mixing the σ70 subunit and the core enzyme in a 2:1 ratio. *S. cerevisiae* Chd1 (residues 118–1,274) was purified from *E. coli* as described previously^42,50^.

### Preparation of DNA constructs

To probe unwinding, a DNA construct was assembled by annealing two partially complementary, modified oligonucleotides (Supplementary Table 2) as described previously^28^. The Widom 601 positioning sequence^51^ was used in nucleosome constructs for homogenous nucleosome positioning. Both nucleosome constructs had asymmetric lengths of flanking DNA (Supplementary Table 1). The DNA for nucleosome assembly was prepared by annealing and ligating (T4 DNA ligase, NEB) a set of complementary, overlapping oligonucleotides (Supplementary Tables 1 and 2), followed by purification using a Mini Prep Cell preparative electrophoresis apparatus (Bio-Rad). Modified and HPLC-purified oligonucleotides were obtained from Integrated DNA Technologies.

### Preparation of fluorophore-labeled mononucleosomes

Nucleosomes were prepared with *Xenopus laevis* histones that were expressed and purified as described previously^52^. Nucleosomes for experiments with T7 RNAP were assembled from labeled DNA and unlabeled histones using the salt gradient dialysis method^52^ and purified using a Bio-Rad Mini Prep Cell apparatus. Nucleosomes for monitoring the entry-side DNA movement during remodeling contained Cy5-labeled H2B(K120C) on the side of the nucleosome adjacent to the short DNA linker (exit side). To achieve homogeneous labeling, these nucleosomes were assembled from oriented hexasomes containing labeled H2A/(H2B-Cy5) dimer^53^. For labeling of H2B, the single-cysteine histone variant H2B(K120C) was reacted with Cy5-maleimide as previously described^54^. H2A/(H2B-Cy5) dimers were refolded from the Cy5-labeled H2B(K120C) and unlabeled H2A and purified by size-exclusion chromatography. Wild-type H3/H4 tetramers were refolded from histones H4 and H3(C110A) and purified by size-exclusion chromatography. Oriented hexasomes were assembled from wild-type H3/H4 tetramers and labeled H2A/(H2B-Cy5) dimers using the standard salt gradient dialysis technique^51^. To maximize the yield of the hexasome species, H2A/(H2B-Cy5) dimers were added to H3/H4 tetramers at an approximately 1.2:1 ratio. Hexasomes were purified to remove other species (namely, nucleosomes and free DNA) using a Bio-Rad Mini Prep Cell apparatus^53^. The purified hexasome pools were used in single-molecule remodeling experiments, upon the addition of unlabeled H2A/H2B dimers to generate the nucleosomes containing a single labeled H2A/(H2B-Cy5) dimer at the exit side^53^.

### smFRET experiments

smFRET experiments were conducted using a custom-built setup (Extended Data Fig. 2) based on a Nikon Eclipse Ti inverted microscope^38,53,55^. To carry out LAGOON experiments, the setup was supplemented with a 360-nm DPSS UV laser with TTL modulation for NTP uncaging (CNI Optoelectronics Tech, UV-F-360-100mW-TTL1kHz) in addition to the 532-nm DPSS laser (Cobolt, 0532-04-01-0150-700) and the 638-nm laser diode (Cobolt, 0638-06-01-0140-100) used for fluorescence excitation. The 360-nm laser was used instead of a more readily available 405-nm laser^56^ to better match the uncaging spectrum of commercially available caged NTPs. The emission from the three lasers was combined using dichroic mirrors and projected onto a spot (approximately 180 × 60 µm^2^) on the slide surface in the TIRF regime using a lens and a prism. The laser power was measured before the prism. The uncaging laser power of 1 mW corresponds to a power density of approximately 10 W cm^−^^2^. Laser intensities were controlled using neutral density (ND) filters (360-nm laser), a combination of a half-wave plate and a polarization beam splitter (532-nm laser), or via the pump current (638-nm laser). The fluorescence emissions from Cy3, Cy5 and Alexa750 were collected with a ×60 water immersion objective (Nikon, CFI Plan Apo VC 60x WI), filtered with ZET532NF (Chroma) and NF03-642E (Semrock) notch filters, spectrally separated by 635-nm (T635lpxr-UF2) and 760-nm (T760lpxr-UF2) dichroic mirrors (Chroma) and projected side by side onto an Andor iXon Ultra 888 EMCCD camera. Data acquisition was controlled using MicroManager^57^. TTL modulation of the 638-nm and the 360-nm lasers and the shutter in the optical path of the 532-nm laser were controlled by an Arduino board to synchronize laser operation with the data acquisition by the CCD camera. For single-turnover experiments, TTL modulation of the 360-nm laser was used to generate uncaging pulses of the desired duration and period. Fluorescence emission time traces were corrected to account for the direct excitation of Cy5 by the 532-nm laser and of Alexa750 by the 638-nm laser, as well as for the bleedthrough from the Cy3 channel into the Cy5 channel and from the Cy5 channel into the Alexa750 channel. Cy3 and Alexa750 signals were scaled to correct for the differences in quantum yields and the detection efficiencies between these dyes and Cy5. Data were analyzed using the Fiji distribution of ImageJ^58^, IDL and MATLAB. FRET plateaus in single-turnover experiments were selected manually (Supplementary Fig. 1). A syringe pump (Harvard Apparatus) was used to exchange solutions in the flow cell. DMNPE-ATP and NPE-GTP were purchased from Invitrogen and Jena Biosciences, respectively.

### Objective-type TIRF smFRET imaging

Objective-type TIRF experiments were carried out on a Nikon Eclipse Ti2-E motorized microscope equipped with a CFI Apochromat TIRF 60XC objective. 532-nm (Cobolt, 0532-04-01-0150-700) and 405-nm (Coherent, Obis-100mW-1284371) lasers were used for fluorescence excitation and uncaging, respectively. Excitation and emission were separated with a multi-band dichroic mirror (Semrock, Di03-R405/488/532/635-t1-25/36). The fluorescence emissions from Cy3 and Cy5 were filtered with a ZET532NF (Chroma) notch filter, spectrally separated by 635-nm (T635lpxr-UF2) dichroic mirrors (Chroma) and projected side by side onto an Andor iXon Ultra 897 EMCCD camera. Data acquisition was controlled using MicroManager.

### Helicase smFRET experiments

The experiments (Fig. 2) were carried out in imaging buffer containing 40 mM Tris pH 7.5, 12 mM HEPES pH 7.9, 60 mM KCl, 0.32 mM EDTA, 3 mM MgCl_2_, 100 µg ml^−1^ of acetylated BSA (Promega), 10% (v/v) glycerol, 10% (w/v) glucose and 2 mM Trolox to reduce photoblinking of the dyes^59^ as well as an enzymatic oxygen scavenging system (composed of 800 μg ml^−1^ of glucose oxidase and 50 μ ml^−1^ of catalase). The T4 helicase concentration was 100 nM in all LAGOON experiments (unless stated otherwise) and 50 nM in regular ATP experiments. For LAGOON experiments, the imaging buffer was supplemented with 10 U ml^−1^ of hexokinase and either 2 (Fig. 2a,b,d) or 10 (Fig. 2c,e,f) mM DMNPE-ATP, and continuous uncaging with a 360-nm laser was used.

### Nucleosome sliding smFRET experiments

The experiments (Fig. 3 and Extended Data Figs. 3 and 4) were carried out in imaging buffer containing 40 mM Tris pH 7.5, 12 mM HEPES pH 7.9, 60 mM KCl, 0.32 mM EDTA, 3 mM MgCl_2_, 100 µg ml^−1^ of acetylated BSA (Promega), 10% (v/v) glycerol, 10% (w/v) glucose and 2 mM Trolox to reduce photoblinking of the dyes as well as an enzymatic oxygen scavenging system (composed of 800 μg ml^−1^ of glucose oxidase and 50 μg ml^−1^ of catalase). The Chd1 concentration was 10 μM in all LAGOON experiments and 1 μM in regular ATP experiments. For LAGOON experiments, the imaging buffer was supplemented with 10 mM DMNPE-ATP and 1 U ml^−1^ of hexokinase.

### T7 RNAP smFRET experiments

T7 RNAP experiments (Fig. 4a–f) were carried out in the buffer recommended by the manufacturer (40 mM Tris-HCl pH7.9, 6 mM MgCl_2_, 1 mM DTT, 2 mM spermidine), supplemented with 10% (w/v) glucose, 2 mM Trolox, 800 μg ml^−1^ of glucose oxidase and 50 μg ml^−1^ of catalase. In LAGOON experiments, the buffer was additionally supplemented with 1 U ml^−1^ of hexokinase and 2 mM DMNPE-ATP and 0.5 mM CTP, GTP and UTP each (Fig. 4d,e) or 1 mM NPE-GTP, ATP, CTP and UTP each (Fig. 4f). The T7 RNAP concentration was 2,500 U ml^−1^ in all experiments.

### AFS

AFS experiments were performed as previously described^46^ with a commercial AFS setup (Lumicks) modified for LAGOON experiments by incorporating a 405-nm uncaging laser (Fig. 4g–i). The uncaging laser power was measured before the objective. The stalled complexes were formed using a DNA matrix and biotinylated *E. coli* RNAP. The DNA matrix encodes the T7 A1 promoter, an 18-nucleotide (nt) dTTP-less sequence (for stalled complex formation), followed by the *E. coli* rpoB gene and terminator sequences rrnB T1 and rrnB T2. The DNA substrate was synthesized by PCR using the pRL574 plasmid as a template and a special 100-nt megaprimer modified with digoxigenin^46^ (Supplementary Table 1). Stalled RNAP–DNA complexes were formed in vitro at 37 °C for 20 minutes (100 nM RNAP and 2 nM DNA matrix in the transcription buffer: 40 mM Tris-HCl, 40 mM KCl, 1 mM DTT, 5 mM MgCl_2_, 35 μM ATP, 35 μM GTP, 35 μM CTP and 500 μM ApU). Stalled complexes were flushed into the AFS chamber with pre-immobilized α-digoxigenin antibodies (Roche). Subsequently, streptavidin-coated beads (Spherotech, 2.1-μm diameter) were introduced into the AFS flow cell in transcription buffer (40 mM Tris-HCl, 80 mM KCl, 0.5 mM DTT, 10 mM MgCl_2_, 0.02% pluronic, 0.02% casein and 50 μg ml^−1^ of heparin). All unbound microspheres were washed out. The necessary calibrations were performed before a 4–6-pN opposing force was applied to the DNA–RNAP-beads tethers. A complete set of nucleotides containing 1 mM GTP, 1 mM CTP, 1 mM UTP and 1 mM of NPE-caged ATP was flown into the chamber and incubated for 2 minutes. Transcription elongation was initiated by switching on the 405-nm laser for 1 minute, switching it off for 1 minute and repeating this sequence several times. The movement of RNAP was detected by the displacement of the microsphere relative to the anchoring point of the DNA tether using the AFS LabVIEW-based software (Lumicks)^17,60^. To generate the elongation rate histograms, the raw data were analyzed using a custom program written in Python. Individual elongation profiles were smoothed with a 10-second median filter, followed by Savitsky–Golay filtering (SG rank = 3 seconds, polynomial order = 3). To evaluate the effect of uncaging on the transcription velocity, average elongation rates over a window of 6.25 seconds were calculated for the intervals of 30 seconds before and after switching on the uncaging laser and plotted as a histogram (Fig. 4i). NPE-ATP was purchased from Jena Biosciences.

### Reporting Summary

Further information on research design is available in the Nature Research Reporting Summary linked to this article.

## Online content

Any methods, additional references, Nature Research reporting summaries, source data, extended data, supplementary information, acknowledgements, peer review information; details of author contributions and competing interests; and statements of data and code availability are available at 10.1038/s41589-022-01100-9.

## Supplementary information

Supplementary Information Supplementary Tables 1 and 2, Supplementary Figs. 1–7 and Supplementary Note 1.

Reporting Summary

## Data Availability

The data supporting the findings of this study are available at 10.17044/scilifelab.20107199.

## Code availability

New codes developed for this publication are available at https://github.com/deindllab/NCHEMB-A220214425A.
