# Supplementary material for: Spatiotemporally controlled generation of NTPs for single-molecule studies
Source: Nat Chem Biol. 2022 Sep 21;18(10):1144–51. doi: 10.1038/s41589-022-01100-9 (PMC9512701; doi:10.1038/s41589-022-01100-9)
Supplement: Supplementary file 1 — Supplementary Tables 1 and 2, Supplementary Figs. 1–7 and Supplementary Note 1. [file 41589_2022_1100_MOESM1_ESM.pdf]

nature portfolio

https://doi.org/10.1038/s41589-022-01100-9

Supplementary information

Spatiotemporally controlled generation of NTPs for single-molecule studies

In the format provided by the authors and unedited

<table>
<tr><td colspan="2">Supplementary Table 1: DNA sequences used to assemble the mononucleosomes described in this manuscript. The TA-rich and TA-poor halves of the 601 positioning sequence are shaded in dark and light grey, respectively.</td></tr>
<tr><td colspan="2">Legend: 601 = “TA-rich side - dyad - TA-poor side”, T7 promoter</td></tr>
<tr><td>Top strand</td><td>Bottom strand</td></tr>
<tr><td colspan="2">1) smFRET construct for monitoring the entry-side DNA movement during nucleosome sliding. Oligonucleotides: F1_new, F2, F3_A750, F4, F5_new, R1_iCy3, R2_new, R3_new, R4_new</td></tr>
<tr><td>5’-Biotin-GGTACCCGTAGATCCTCTAGAGTGGGAGCTCGGAACACTATCCGACTGGCAC
CGGCAAGGTCGCTGTTCAATACATGCACAGGATGTATATATCTGACACGTGCCTGGAGACTA
GGGAGTAATCCCCTTGGCGGTTAAAACGCGGGGGACAGCGCGTACGTGCGTTTAAGCGGTGC
TAGAGCTGTCTACGACCAATTGAG-iCy3-GGCCTGCAGACCGGGATTCTCCAGGGC-3’</td><td>5’-GCCCTGGAGAATCCCGGTCTGCAGGCCACTCAATTGGTCGTAGACAGCTCTAGCAC
CGCTTAAACGCACGTACGCGCTGTCCCCCGCGTTTTAACCGCCAAGGGGATTACTCCCT
AGTCTCCAGGCACGTG-T-Alexa750-CAGATATATACATCCTGTGCATGTATTGAAC
AGCGACCTTGCCGGTGCCAGTCGGATAGTGTTCCGAGCTCCCACTCTAGAGGATCTACG
GGTACC-3’</td></tr>
<tr><td colspan="2">2) smFRET construct for monitoring nucleosome unwrapping upon its invasion by the T7 RNA polymerase. Oligonucleotides: F1_new_Cy5, F2, F3, F4, F5_T7, R1_new, R2_new, R3_new_Cy3, R4_T7</td></tr>
<tr><td>5’-Biotin-GGTACCCGTATAATACGACTCACTATAGGGCGGTTCTCTTTCCGTCTGGCTC
CGGCAAGGTCGCTGTTCAATAC/iCy3/TGCACAGGATGTATATATCTGACACGTGCCTGGA
GACTAGGGAGTAATCCCCTTGGCGGTTAAAACGCGGGGGACAGCGCGTACGTGCGTTTAAGC
GGTGCTAGAGCTGTCTACGACCAATTGAGTGGCCTGCAGACCGGGATTCTCCAGGGC-3’</td><td>5’-Cy5-GCCCTGGAGAATCCCGGTCTGCAGGCCACTCAATTGGTCGTAGACAGCTCTA
GCACCGCTTAAACGCACGTACGCGCTGTCCCCCGCGTTTTAACCGCCAAGGGGATTACT
CCCTAGTCTCCAGGCACGTGTCAGATATATACATCCTGTGCATGTATTGAACAGCGACC
TTGCCGGTGCCAGTCGGATAGTGTTCCGCCCTATAGTGAGTCGTATTATACGGGTACC-
3’</td></tr>
<tr><td colspan="2">3) Digoxigenin modified megaprimer used together with the F900 primer for generating the construct for acoustic force spectroscopy. Primers: F100 and R100</td></tr>
<tr><td>CATATCTACAAGCCATCCCCCCACAGATACGGTAAACTAGCCTCGTTTTTGCATCAGGAAAG
CAGAAGCTTGGCGTAATCATGGTCATAGCTGTTTCCTG</td><td>CAGGAAACAGCTATGACCATGATTACGCCAAGCTTCTGCTTTCCTGATGCAAAAACGAG
GCTAGTTTACCGTATCTGTGGGGGGATGGCTTGTAGATATG</td></tr>
</table>

**Supplementary Table 2:** Oligonucleotide sequences, used for generating DNA constructs.	
**Name**	**Sequence**
F1_new	GCCCTGGAGAATCCCGGTCTGCAGGCCACTCAATT
F2	/5phos/GGTCGTAGACAGCTCTAGCACCGCTTAAACGCACGTACGCGCTGTC
F3	/5Phos/CCCCGCGTTTTAACCGCCAAGGGGATTACTCCCTAGTCTCCAGGCACGTGTCAGATATATAC
F3_A750	/5phos/CCCCGCGTTTTAACCGCCAAGGGGATTACTCCCTAGTCTCCAGGCACGTG/iAlex750N/CAGATATATAC
F4	/5phos/ATCCTGTGCATGTATTGAACAGCGACCTTGCCGGTGCCAGTCGGAT
F5_new	/5phos/AGTGTTCCGAGCTCCCACTCTAGAGGATCTACGGGTACC
F5_T7	/5phos/AGTGTTCCGCCCTATAGTGAGTCGTATTATACGGGTACC
R1_new	/5phos/CGGTGCTAGAGCTGTCTACGACCAATTGAGTGGCCTGCAGACCGGGATTCTCCAGGGC
R1_iCy3	/5phos/CGGTGCTAGAGCTGTCTACGACCAATTGAG/iCy3/GGCCTGCAGACCGGGATTCTCCAGGGC
R2_new	/5phos/TAATCCCCTTGGCGGTTAAAACGCGGGGGACAGCGCGTACGTGCGTTTAAG
R3_new	/5phos/GTCGCTGTTCAATACATGCACAGGATGTATATATCTGACACGTGCCTGGAGACTAGGGAG
R3_new_Cy3	/5phos/GTCGCTGTTCAATAC/iCy3/TGCACAGGATGTATATATCTGACACGTGCCTGGAGACTAGGGAG
R4_new	/5BiotinTEG/GGTACCCGTAGATCCTCTAGAGTGGGAGCTCGGAACACTATCCGACTGGCACCGGCAAG
R4_T7	/5BiotinTEG/GGTACCCGTATAATACGACTCACTATAGGGCGGAACACTATCCGACTGGCACCGGCAAG
10AT30GC-Cy3	TTTTTTTTTTTTTTTTTTTTTTTTTTTTTT/iCy3N/ATTATTATTTGCGGGGCGGGCGGGGCGGCGGGCGCGGGCG
10AT30GC-Cy5	/5BioTEG/CGCCCGCGCCCGCCGCCCCGCCCGCCCCGCAAATAATAA/iCy5N/TTTTTTTTTTTTTTTTTTTTTTTTTTTTTT
F100	CATATCTACAAGCCATCCCC
R100	CAGGAAACAGCTATGACCAT
F900	CCGAACGCCTATCTTAAAGTTTAAACATAAAGACCAGACC

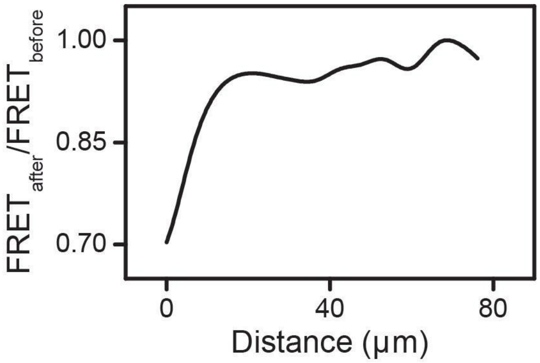

Supplementary Figure 1: Distribution of the extent of DNA unwinding as a function of the distance from the edge of the uncaging area. The data represent the average of three FOVs on the same slide. The assay was conducted in the presence of 200 nM T4 helicase, 10 mM DNMPE-ATP and 10 U/ml hexokinase. Uncaging was limited to ~1/3 of the FOV with a sharp edge (along the y-axis). The extent of unwinding after uncaging for 1 min with the 405-nm laser at ~20 W/cm^2 was estimated as the ratio between FRET signals after and before uncaging and plotted as a function of the distance from the edge. The experiment was carried out on an objective-type TIRF set-up that enables precise control over which parts of FOV are illuminated.

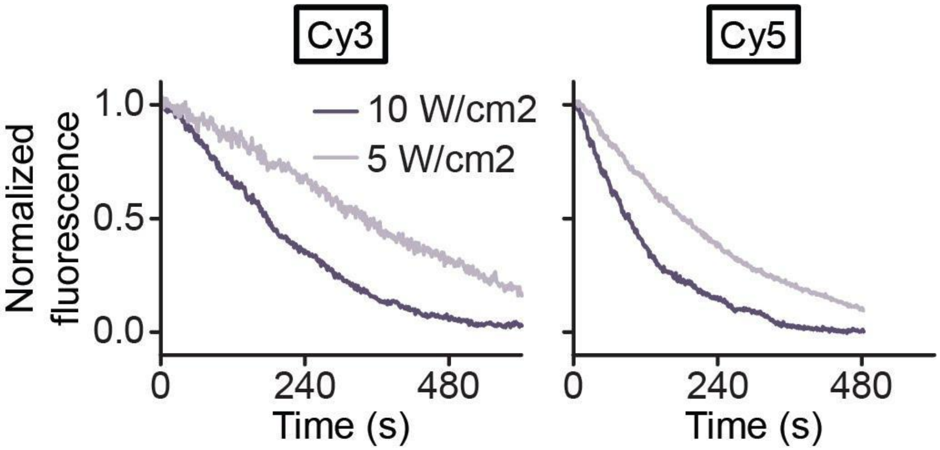

Supplementary Figure 2: The effect of the uncaging 360-nm laser on Cy3 and Cy5 photobleaching. Normalized fluorescence intensity of Cy3 (left) and Cy5 (right) as a function of time during continuous exposure to the 360-nm light with a power density of approximately 10 or 5 W/cm^2 in the presence of 1 mM DMNPE-ATP. The measurement was conducted using the helicase construct.

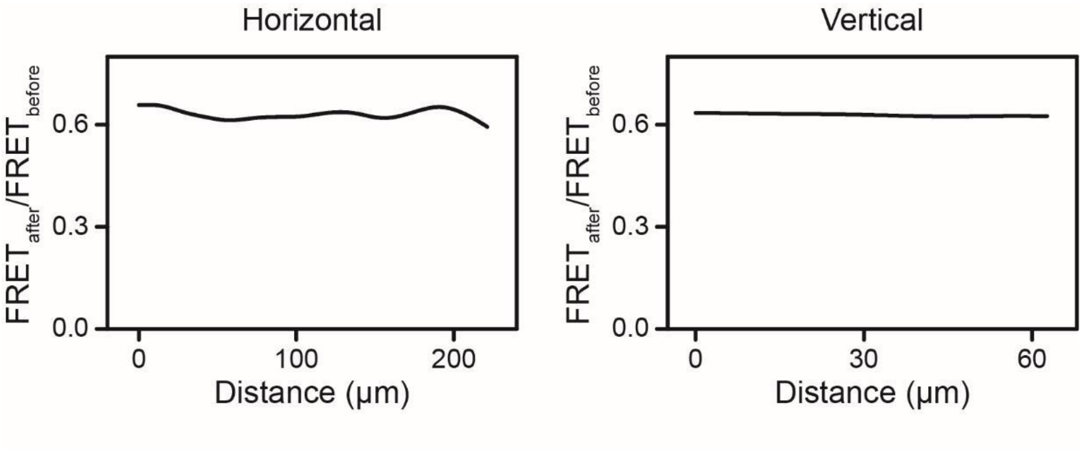

Supplementary Figure 3: Distributions of the ratio between Cy5 fluorescence intensities upon 532-nm excitation (FRET) after and before uncaging for 1 min with 1 W/cm^2 360-nm laser in the horizontal (x, left) and vertical (y, right) directions throughout a FOV. The smFRET LAGOON experiment to monitor DNA unwinding was carried out in the presence of 200 nM T4 hexokinase, 2 mM DMNPE-ATP and 10 U/ml hexokinase. The ratio between FRET after and before uncaging reports on the extent of unwinding. The data represent the average from three FOVs on the same slide.

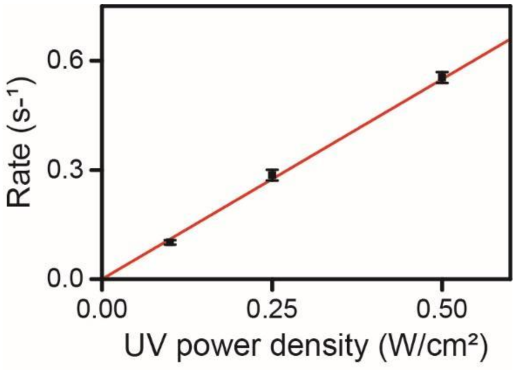

Supplementary Figure 4: DNA unwinding rates (mean±s.e.m. and a linear fit of the data) as a function of uncaging laser power density in the presence of 200 nM T4 helicase, 10 mM DMNPE-ATP and 1 U/ml hexokinase (*N*=136, 322 and 722).

Supplementary Note 1. Numerical simulations of the uncaging process

To better characterise the uncaging process, especially in the pulsed uncaging regime where an experimental characterization is challenging, we have conducted numerical simulations of the uncaging process. Since the uncaging area (approximately 180 x 60 μm^2 in the smFRET setup) is much larger than the penetration depth of the uncaging laser (<1 μm), ATP can leave the uncaging area predominantly in one direction (away from the surface, z-axis). The process therefore can be well approximated as a 1D diffusion (with the distance from the glass surface as the only coordinate, z). Three main

processes determine the resulting ATP concentration: uncaging, diffusion, and ATP hydrolysis by the hexokinase. The uncaging reaction can be expressed as:

$$cA \underset{1-QY}{\overset{hv}{\longleftrightarrow}} cA^* \xrightarrow{QY} A^{rd} \xrightarrow{k_{unc}} A$$

where cA and cA* are caged ATP in the ground and excited states, respectively, A^{rd} is the rate-determining intermediate of the uncaging reaction, A is ATP, QY is quantum yield and k_{unc} is the uncaging rate constant. The excited state lifetimes for caging groups are in the low-ns range[2], which is much shorter than the average excitation time under the power densities that are relevant for LAGOON (>1 ms at 100 W/cm^2). For this reason, the depletion of the ground state is negligible and the uncaging reaction can be simplified to:

$$cA \xrightarrow{hv*QY} A^{rd} \xrightarrow{k_{unc}} A$$

10% glucose is saturating for the hexokinase, so the ATP hydrolysis rate can be determined as:

$$\frac{\partial A}{\partial t} = -\frac{v_{max} \cdot A}{A + K_m}$$

, where A is the concentration of ATP, v_{max} = 170 μM/s at 10 U/ml hexokinase (based on the definition of a unit), K_m = 300 μM[3]. The diffusion coefficient for ATP (the same is assumed for the caged ATP and the rate-determining intermediate) is approximately 3.7 x 10^{-6} cm^2/s in water[4], which corresponds to approximately 2 x 10^{-6} cm^2/s in the imaging buffer that contains glycerol and glucose. The concentrations of DMNPE-ATP (cA), the rate-determining uncaging intermediate (A^{rd}), and ATP (A) therefore follow:

$$\frac{\partial A}{\partial t} = D\frac{\partial^2 A}{\partial z^2} + k_{unc} \cdot A^{rd} - \frac{v_{max} \cdot A}{A + K_m}$$

$$\frac{\partial A^{rd}}{\partial t} = D\frac{\partial^2 A^{rd}}{\partial z^2} - k_{unc} \cdot A^{rd} + QY \cdot \sigma \cdot \Phi \cdot e^{-\frac{z}{d}} \cdot cA$$

$$\frac{\partial cA}{\partial t} = D\frac{\partial^2 cA}{\partial z^2} - QY \cdot \sigma \cdot \Phi \cdot e^{-\frac{z}{d}} \cdot cA$$

, where D is the diffusion coefficient, k_{unc} is the uncaging rate (18 s^{-1} for DMNPE-ATP[2]), v_{max} = 170 μM/s at 10 U/ml hexokinase (based on the definition of a unit), K_m = 300 μM[3], QY is the uncaging quantum yield (7% for DMNPE-ATP[2]), σ is the absorption cross-section for caged ATP (1.9 x 10^{-17} cm^2, for DMNPE-ATP, corresponding to the molar extinction coefficient of 5000 $M^{-1}cm^{-1}$ (2)), Φ is the photon flux for the uncaging light at the glass-buffer interface (approximately 1.8 x 10^{20} $cm^{-2}s^{-1}$ for the maximum uncaging power density of 100 W/cm^2 used for pulsed uncaging experiments), the exponential term $e^{-z/d}$ represents the decay of the evanescent field upon total internal reflection, where d is the penetration depth as determined using the following equation:

$$d = \frac{\lambda}{4\pi\sqrt{n_1^2 \cdot sin^2\alpha - n_2^2}}$$

, where λ is the uncaging light wavelength (360 nm), α is the TIRF angle (~76° for the prism-based TIRF smFRET setup), n_1 and n_2 are the refractive indices of quartz (1.46) and imaging buffer (~1.36), respectively. The penetration depth therefore is ~70 nm. A Matlab script (available at https://github.com/deindllab/NCHEMB-A220214425A) was used to solve this system of differential equations numerically in the range from 0 to 20 μm in z and 0 to 5 s in t, with the following initial and boundary conditions:

$$A = A^{rd} = 0,\ cA = cA_0\ when\ t = 0$$

$$\frac{\partial A}{\partial z} = \frac{\partial A^{rd}}{\partial z} = \frac{\partial cA}{\partial z} = 0\ when\ z = 0$$

$$A = A^{rd} = 0,\ cA = cA_0\ when\ z = 20\ \mu m$$

, where cA_0 is the initial concentration of caged ATP (2 or 10 mM for DMNPE-ATP).

First we analyzed the steady-state ATP concentrations achieved with continuous uncaging (Supplementary Fig. 5a,b). As expected, the steady-state ATP concentration increases linearly with increasing uncaging power density for the whole range used for continuous uncaging in this study. The ATP concentrations derived from our simulations are approximately 2.5-fold higher than the ones determined experimentally (Fig. 2), which is well within the expected range, given that many parameters from the literature could deviate from those under the specific experimental conditions used in our study (e.g., the uncaging quantum yield). Since the goal of the simulation is to analyze trends rather than to obtain accurate absolute values, we refrained from introducing any correction factors (e.g., an apparent uncaging quantum yield) to achieve an even better quantitative agreement between the model and the experimental data. The numerical

simulations highlighted the TIRF angle as an important parameter that affects the resulting ATP concentrations (Supplementary Fig. 5b). While photobleaching and photodamage are proportional to the uncaging light intensity at z = 0, the ATP concentration near the surface increases with increasing penetration depth. It is therefore beneficial to adjust the TIRF angle close to the critical angle for LAGOON experiments, since this would allow the same ATP concentrations to be achieved at lower uncaging light intensities, and thus minimize photodamage and photobleaching.

Next, we simulated the pulsed uncaging regime (Supplementary Fig. 5c,d). The peak ATP concentration scaled almost linearly with the pulse duration throughout the 30-180 ms range used in the study (Supplementary Fig. 5d). The simulations demonstrate that the ATP concentration decays with a half-time of approximately 400 ms after the pulse is over (Supplementary Fig. 5c). Thus, ATP diffusion under the widefield uncaging conditions limits the minimal duration of the ATP pulse. Diffusion is relatively slow because with a large uncaging area, ATP can leave the FOV predominantly in one direction only - away from the surface (z). For this reason, achieving single-turnover conditions with ATPases that are faster than Chd1 would require additional measures. For example, slowing down the ATP hydrolysis rate (e.g., by using a different divalent ion, like Ca^{2+}, or a sub-saturating divalent ion concentration, or both), or switching to uncaging within a diffraction-limited spot. The latter would dramatically speed up the ATP diffusion rate (to the sub-ms timescale[5]) by effectively switching from 1D to 3D diffusion. Alternatively, the duration of the ATP pulse can be shortened by increasing the hexokinase concentration. Moreover, the real rate of ATP diffusion is faster due to the limited size of the uncaging area (as opposed to an infinitely large area in a 1D-diffusion approximation), and the difference increases when the uncaging area decreases in size. The uncaging beam spot size therefore can be used to tune the ATP diffusion rate.

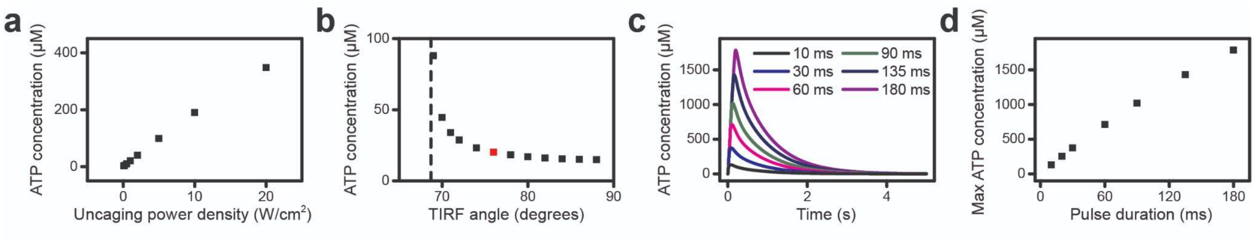

Supplementary Figure 5: Steady-state ATP concentration as a function of uncaging laser power density (**a**) or TIRF angle (**b**) in the presence of 2 mM DMNPE-ATP and 10 U/ml hexokinase. The TIRF angle in (**a**) is 76° (the value for the prism-type TIRF setup used in the study, highlighted in red in (**b**)), the power density in (**b**) is 1 W/cm^2. The dashed line in (**b**) indicates the critical angle. **c**, ATP concentrations near the surface as a function of time for uncaging pulses of different durations. **d**, maximum ATP concentration after an uncaging pulse as a function of pulse duration. Simulation parameters in (**c**) and (**d**) correspond to the conditions used for pulsed uncaging experiments (100 W/cm^2 uncaging power density, 10 mM DMNPE-ATP and 1 U/ml hexokinase).

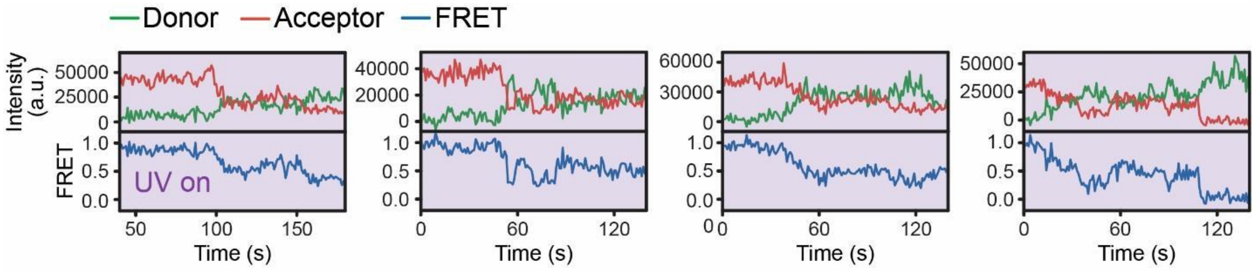

Supplementary Figure 6: Representative smFRET trajectories capturing DNA unwinding by the T4 helicase upon uncaging (1 W/cm^2, 360 nm) in the presence of 100 nM T4 helicase, 2 mM DMNP-EDTA (Biotium), 500 µM $CaCl_2$, and 100 µM ATP (out of N = 190 traces).

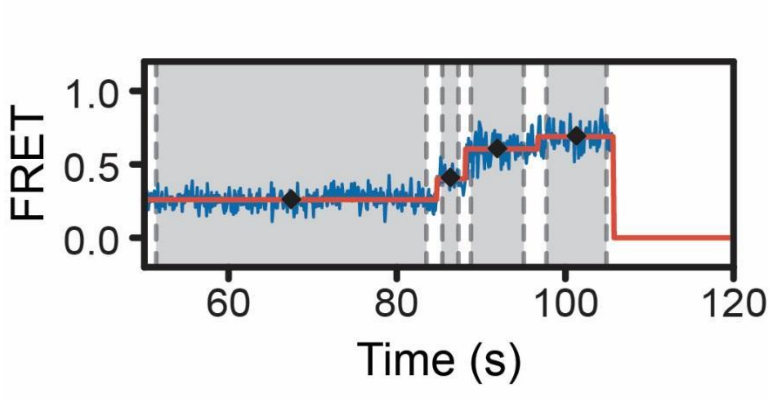

Supplementary Figure 7: Determining plateau FRET values in pulsed uncaging traces. FRET (blue) time trace showing entry-side DNA movement during nucleosome sliding in the pulsed uncaging regime in the presence of 10mM DMNPE-ATP. The red line represents the steps located in the data using the AutoStepFinder[1] method. The grey vertical lines mark manually selected plateau regions (shaded in light grey). The black diamonds represent mean FRET values for manually-selected plateaus. The trace from Fig. 2c (left) is used as an example.

1. Loeff, L., Kerssemakers, J. W. J., Joo, C. & Dekker, C. : A fast and automated step detection method for single-molecule analysis. *Patterns (N Y)* **2**, 100256 (2021).
2. Ellis-Davies, G. C. R. Caged compounds: photorelease technology for control of cellular chemistry and physiology. *Nat. Methods* **4**, 619–628 (2007).
3. Gazith, J., Schulze, I. T., Gooding, R. H., Womack, F. C. & Colowick, S. P. Multiple forms and subunits of yeast hexokinase. *Ann. N. Y. Acad. Sci.* **151**, 307–331 (1968).
4. Hubley, M. J., Locke, B. R. & Moerland, T. S. The effects of temperature, pH, and

magnesium on the diffusion coefficient of ATP in solutions of physiological ionic strength. *Biochim. Biophys. Acta* **1291**, 115–121 (1996).

5. Borejdo, J. & Akopova, I. Orientational changes of crossbridges during single turnover of ATP. *Biophys. J.* **84**, 2450–2459 (2003).
